# CT manifestations of the coronavirus disease 2019 of imported infection versus second-generation infection in patients outside the original district (Wuhan, China) of this disease: An observational study

**DOI:** 10.1097/MD.0000000000020370

**Published:** 2020-05-22

**Authors:** Yu-ping Wu, Jin-ming Cao, Tian-wu Chen, Rui Li, Feng-jun Liu, Yue Zeng, Xiao-ming Zhang, Qi-wen Mu, Hong-jun Li

**Affiliations:** a Sichuan Key Laboratory of Medical Imaging and Department of Radiology, Affiliated Hospital of North Sichuan Medical College; b Department of Radiology, Nanchong Central Hospital / Second School of Clinical Medicine, North Sichuan Medical College; c Department of Infectious Disease, Affiliated Hospital of North Sichuan Medical College, Nanchong, Sichuan; d Department of Radiology, Beijing YouAn Hospital, Capital Medical University, Beijing, China.

**Keywords:** coronavirus, infection, pneumonia, tomography, X-ray computed

## Abstract

To explore the discrepancy in computed tomography (CT) manifestations of the coronavirus disease 2019 (COVID-19) in patients outside the original district (Wuhan, China) between cases with imported infection and second-generation infection, 22 patients with COVID-19 from 2 hospitals in Nanchong, China, 938 km away from the original district (Wuhan, China) of this disease were enrolled. All patients underwent initial and follow-up CT after admission during the treatment, and were divided into 2 groups. Group A and B were composed of 15 patients with a history of exposure to the original district (Wuhan, China) in short-term (i.e., imported infection), and 7 with a close contact with the patients with confirmed COVID-19 or with the healthy individuals from the original district (i.e., second-generation infection), respectively. Initial CT features including extent score and density score between groups were statistically compared. We found that all patients in group A and 3 of 7 patients in group B had abnormal CT findings while 4 of 7 patients in group B had not. Patients with abnormal CT findings were more frequent in group A than in group B (*P* < .05). On initial CT, pure ground glass opacity (GGO), and GGO with consolidation and/or other abnormalities were found in 20% (3/15) and 80% (12/15) patients in group A, respectively, while 1 (14.3%), 2 (28.6%), and 4 (57.1%) had pure GGOs, GGO with focal consolidation, and normal CT appearances in Group B, respectively. Patients with extent and density scores of ≥5 were more frequent in group A than in group B (all *P*-values < .01). Additionally, 3 of 4 (75%) patients with normal initial CT findings had focal pure GGO lesions on follow-up. In conclusion, COVID-19 in patients with a history of exposure to the original district can be severer than with the second-generation infection on CT.

## 1 Introduction

In late December 2019, several medical institutions in Wuhan admitted a cluster of patients with pneumonia of unexplained etiology.^[1–2]^ An unknown novel coronavirus, which was temporarily named as the 2019 novel coronavirus (2019-nCoV), was identified as the pathogen.^[3]^ Subsequently, the pneumonia caused by the 2019-nCoV has been officially named by the World Health Organization as the coronavirus disease 2019 (COVID-19).^[4]^ Accompanied with the arrival of the Chinese Spring Festival travel rush, the 2019-nCoV spread rapidly all over China.^[5]^ As of February 17, 2020, a total of 72,436 patients with confirmed COVID-19 including 42,752 (59.0%) in Wuhan have been reported in China.^[6–7]^ Of these, 11,741 and 1868 cases were severe and death cases, respectively. Furthermore, most of severe (9222, 78.5%) and death (1381, 73.9%) population were from Wuhan, China, the original district of this disease. The rate of severity and mortality in COVID-19 patients from Wuhan were significantly higher than that in other regions outside Wuhan, suggesting patients infected in the original district (Wuhan, China) of this disease may have more rapid aggravation.

To detect COVID-19, viral nucleic acid detection using real-time polymerase chain reaction (RT-PCR) remains the standard of reference.^[8]^ However, several defects such as immature development of nucleic acid detection technology, variation in detection rate from different manufacturers, false negative caused by low patient viral load or improper clinical sampling may cause low efficiency of detection and limit its clinical application.^[8]^ As a promising method recommended by Chinese Society of Radiology,^[9]^ computed tomography (CT) plays an essential role in diagnosis and monitoring treatment responses in COVID-19. Multifocal bilateral ground glass opacity (GGO) as an indicator of early disease stage, and patchy consolidations as a marker of the disease progression are the most common patterns of CT abnormalities.^[9–11]^ Based on above-mentioned typical CT findings, the severity of COVID-19 could be staged into early, progression, severe, and dissipation stage which embodied in the consensus of Chinese Society of Radiology.^[9]^

As for the patients outside the original district (Wuhan, China) of this disease, the infection routes included an exposure history of the original district (Wuhan, China) in short term (i.e., imported infection), and a close contact with the infected individuals exposed to the original district recently (i.e., second-generation infection). To the best of our knowledge, there were no reports focusing on the discrimination in severity of the COVID-19 between patients outside the original district (Wuhan, China) according to different routes of infection. Thus, the purpose of our research was to determine the discrepancy in CT manifestations of COVID-19 in patients outside the original district (Wuhan, China) between cases with imported infection and with second-generation infection, aiming to help clinicians outside the original district formulate more accurate and effective prevention and treatment measures.

## 2 Materials and methods

### 2.1 Patients

The institutional ethics committee of the Affiliated Hospital of North Sichuan Medical College approved this study (approval number, 2020ER007–1), and the written informed consent was obtained from each participant.

From January 23 to February 17, 2020, 22 consecutive COVID-19 patients derived from 2 designated hospitals in Nanchong, China, 938 km away from the original district (Wuhan, China) of this disease, were enrolled into our study. All patients had positive results for 2019-nCoV detection via the initial RT-PCR after admission. Patients were subsequently classified into 2 groups based on the following criteria:

(1) patients with imported infection were enrolled into group A, and they had a history of travelling to/or living in the original district (Wuhan, China) recently for less than 1 month.
(2) in group B, patients had second-generation infection, and they were in the absence of exposure to the original district (Wuhan, China) but were in close contact with the patients with confirmed COVID-19, or with the healthy individuals from the original district (Wuhan, China).

The baseline data of the onset of symptoms are recorded in Table 1.

**Table 1:**
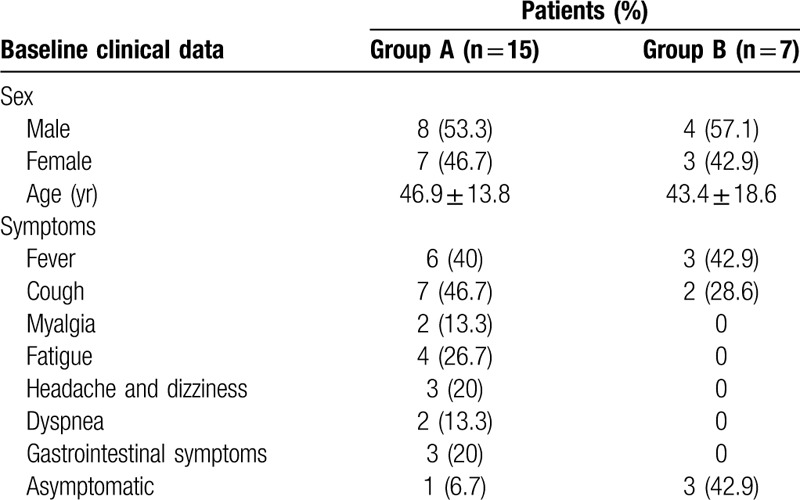
Baseline clinical characteristics of the coronavirus disease 2019.

**Figure 1:**
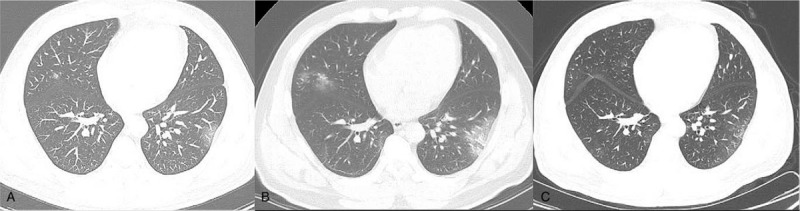
In a 46-year-old male with coronavirus disease 2019 by imported infection, the findings on initial computed tomography (CT) (day 1 after admission) are multiple subpleural focal ground glass opacities (GGOs) in both lungs (A). On day 8, the lesions manifest as larger-scale GGOs with great consolidation in some previous GGOs in both lungs on the follow-up CT images (B). On day 12, the ranges of lesions have reduced in a great extent, and CT images show strip-like opacity (C).

**Figure 2:**
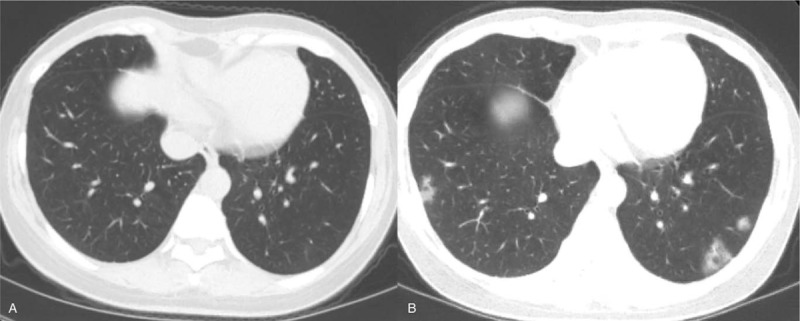
In a 39-year-old asymptomatic female with coronavirus disease 2019 by second-generation infection, no abnormal findings have been found on initial computed tomography scan after admission (A) but positive nucleic acid test on admission. On the follow-up computed tomography on day 8 after admission, focal patchy ground glass opacities appear in the lower lobes of both lungs (B).

All patients underwent initial thoracic CT examinations (Fig. 1A andFig. 2A) after admission. The intervals between the initial CT scan and the onset of symptoms were 4.9 ± 3.9 days in group A and 10 ± 4.5 days in group B. It should be noted that 1 patient in group A, and 3 in group B were asymptomatic, and there was no interval between the initial CT scan and the onset of symptoms. All patients underwent follow-up CT scans (Fig. 1B and C, and Fig. 2B) and RT-PCR every 3 to 8 days during their hospitalization based on the severity of COVID-19. But for the asymptomatic patients in group B, they received follow-up CT scans when their RT-PCR results were positive. In addition, all patients received relevant medical management during their hospitalization.

### 2.2 Image acquisition

Thoracic non-contrast enhanced CT scans were performed in 17 patients with 16-row multidetector row CT system (uCT 510, United Imaging, Shanghai, China), and in 5 patients with a 128-row multidetector CT system (SOMATOM Definition Flash, Siemens Healthcare systems, Germany). Each examination was performed in a breath-hold mode at full suspended inspiration. The scanning coverage was from the thoracic inlet to the middle level of the left kidney. Scanning parameters for the uCT 510 scanner were as follows:

- tube voltage of 120 KV
- tube current of 200 mA (automatic exposure control employed)
- rotation time of 0.35 s, pitch of 1.5 mm
- detector collimation of 0.625 mm
- slice thickness / reconstruction thickness of 5 / 1 mm

The scanning parameters for SOMATOM Definition Flash scanner were similar to those for the 16-multidetector row CT scanner except the tube current of 250 mA and detector collimation of 0.6 mm. Data from 2 CT scanners were respectively transferred to the image processing workstation (SOMATOM Definition Flash, Siemens Healthcare systems, Germany). The window width and level were set to 350 HU and 40 HU for mediastinal window, and to 1000 HU and -700 for lung window, respectively.

### 2.3 CT data analysis

All image data were independently reviewed on above-mentioned workstation by 2 experienced radiologists (the first author with 1 year of experience in radiology and the co first author with 8 years of experience in radiology) blinded to epidemiologic and clinical information. In case of discrepancy between the 2 observers, a third radiologist (co corresponding author with 12 years of experience in radiology) reviewed the images for the final adjudication. Before the previous radiologists reviewed the image data, a professor of radiology (the corresponding author with 22 years of experience in body radiology) trained them on how to review the image data.

According to the expert consensus,^[9]^ the initial CT manifestations in groups A and B were assessed based on the following features:

(1) no abnormal finding
(2) GGO
(3) consolidation
(4) other abnormalities (e.g., reticulation, and interlobular septal thickening)

In order to assess the severity of the disease more accurately, we also devised a semi-quantitative scoring system to evaluate the extent and severity of disease in this study. As illustrated in Tables 2 and 3, the CT lesion extent and density scores were determined based on the anatomic distribution and density of lung lesions referencing to the reported semi-quantitative score system.^[12]^ The extent score was assessed on lung window based on the extent of the 5 lung lobes involved by COVID-19. The overall lung extent score was obtained by summing the 5 lobe scores. The density score was evaluated on lung window based on the percentages of consolidation and other abnormalities in each COVID-19 lesion, and the overall lung density score was acquired by summing the 5 lobe scores. The score range for both lungs in each patient is from 0 (no detectable abnormality) to 20 (more than 75% of each lung lobe involved by COVID-19 lesion and 100% of consolidation in each lesion).

**Table 2:**
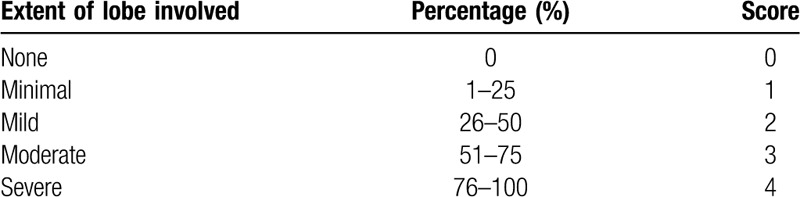
The extent scoring system of coronavirus disease 2019 on computed tomography.

**Table 3:**
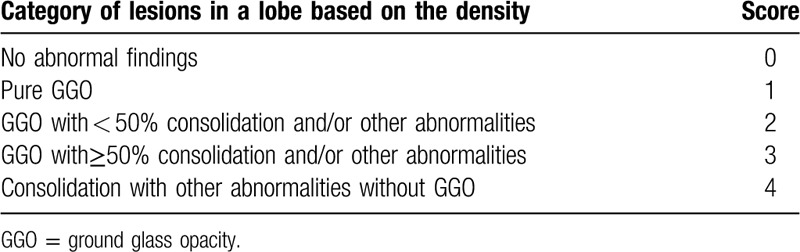
The density scoring system of the coronavirus disease 2019 on initial computed tomography.

In order to assess the intra-observer variability of the above semi-quantitative measurements, the first author repeated the image data analysis 3 days later. The intra-observer variability was obtained by comparison of the 2 measurements by the first author. The inter-observer variability was accessed with the results by 2 independent double-blinded observers (the first author and the co-first author).

### 2.4 Statistical analysis

All data were statistically analyzed by IBM SPSS statistics software (version 25.0 for Windows; SPSS, Chicago, IL, USA). The normality of distribution was evaluated by Shapiro–Wilk test. Continuous variables, expressed as the mean and standard deviation. The categorical variables were described in percentiles and compared using the Chi-squared test or Fisher exact test. Both intra-observer and inter-observer variability were tested for CT score using inter-class correlation coefficient (ICC). The semi-quantitative extent and density scores of COVID-19 lesions on initial CT were considered to be reproducible when the ICC was greater than 0.75.^[13]^ Statistical difference was defined as *P* < .05 for all tests.

## 3 Results

### 3.1 CT manifestations

15 (100%) patients in Group A (Fig. 1A) and 3 (42.9%) in group B had abnormal findings on initial CT while the remaining 4 (57.1%) patients had none abnormal CT findings. Patients with abnormal CT findings were more frequent in group A than in group B (*P* < .05). Pure GGOs, and GGOs with consolidation and/or other abnormalities were observed in 3 (20%) and 12 (80%) patients in group A, respectively. In group B, 1 (14.3%) and 2 (28.6%) patients had pure GGOs and GGOs with consolidation, respectively. Among the previous 4 patients with normal image on initial CT scan, 3 cases (75%) developed into focal pure GGO on follow-up scans (Fig. 2A).

### 3.2 Quantification of CT appearance

The mean intra-observer and inter-observer ICC values were 0.96 (95%CI: 0.91–0.98) and 0.94 (95%CI: 0.86–0.97) for extent score, and 0.95 (95%CI: 0.90–0.98) and 0.93 (95%CI: 0.84–0.97) for density score, respectively. Therefore, the average of the extent score and density score from the first author and the co first author's measurements was used for the subsequent statistical analysis.

As demonstrated in Table 4, the mean extent score of lesions on CT in group A was 6.7, ranged from 1 to 17. In group B, the mean extent score of lesions on CT was 1.1, ranged from 0 to 4. In group A, 13 (86.7%) and 2 (13.3%) patients scored at least 5 and 10, respectively. Except the 4 patients with a normal CT finding on initial scans, the remaining 3 patients in group B scored 1, 3, and 4 according to the extent scoring system. The extent of lung lobe involved by COVID-19 lesions in group A was strikingly greater than that observed in group B (*P* < .001).

**Table 4:**
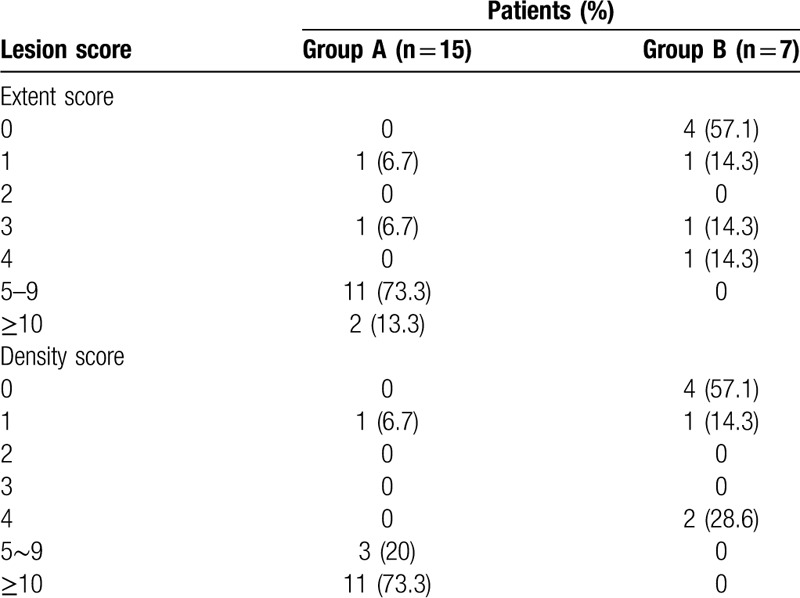
Comparisons of patients between groups according to the extent and density scores of the coronavirus disease 2019 on initial computed tomography.

As shown in Table 4, the mean density score of lesions in both lungs in group A (mean score, 10.1; range, 1–17) was significantly higher than that in group B (mean score, 1.3; range, 0–4). In group A, 14 and 11 patients scored at least 5 (93.3%) and 10 (73.3%), respectively. In contrast, none of patients in group B with abnormal CT findings on initial scans had lesion density score of more than 5 (*P* < .001), indicating that the COVID-19 in second-generation infected patients could be milder when compared with those with imported infection.

## 4 Discussion

COVID-19 is a new disease with high infectivity causing an enormous impact on public health.^[14]^ The Chinese Spring Festival travel rush has triggered massive population movements which gave rise to the confirmed cases of COVID-19 outside the original district (Wuhan, China) of this disease with imported infection, as well as cases with second-generation infection in succession. In order to better master the characteristics of COVID-19 in patients outside the original district for appropriate treatment, we carried out our study to investigate the discrepancy in CT manifestations of this pneumonia in patients outside the original district (Wuhan, China) between cases with imported infection and the second-generation infection.

Our study revealed that abnormal findings on initial CT scans can be found in each patient with imported infection but not patients with the second-generation infection. In patients with the second-generation infection, some patients could have abnormal initial CT appearances, and some could not. Our findings can be explained by the following pathological mechanism. As reported,^[15–17]^ RNA virus is characterized by error-prone viral replication and recombination and usually generates progeny viruses with highly diverse genomes which might result in reduction of virulence and pathogenicity. We could presume that the 2019-nCoV as a novel RNA virus might have the similar characteristics of reduction of virulence and pathogenicity resulted from the error-prone viral replication and recombination.

As shown in our study, GGO and consolidation could be the most common patterns of CT abnormalities of the confirmed COVID-19 in patients with imported infection, which was consistent with the published reports.^[9–11]^ As reported,^[9]^ GGO and consolidation could respectively reflect the potential pathological abnormalities in different stages of the disease. Seen mainly in the early stage of the disease, the underlying pathologic change of pure GGO can be small amount of exudation of fluid in alveolar cavity and interlobular interstitial edema.^[10]^ Consolidation lesions could be regarded as a marker of more severe phase,^[11]^ reflecting a large amount of cell-rich or fibrous exudation accumulated in the alveolar cavity and pulmonary interstitium.^[10]^ It is noteworthy that 3 cases of second-generation with normal finding on initial CT scan developed into focal GGOs during follow-up CT, suggesting that the limitation of CT in the early detection of asymptomatic patients with the second-generation. The COVID-19 case without abnormal manifestation on initial CT scan should be confirmed by 2019-nCoV detection via RT-PCR together with a history of close contact with imported infection.

Moreover, we found that the discrepancies of extent and density scores obtained on the initial CT could exist between patients with imported infection and with the second-generation infection. In detail, the extent of lung lobe involved by COVID-19 lesions in patients with imported infection was strikingly greater than that in patients with second-generation infection. The previous discrepancies of extent and density scores between groups can be explained as follows. On one hand, the virus load or the chance of being exposed to the virus in the environment in the original district (Wuhan) could be much higher than any other district where there were much fewer COVI-19 cases. On the other hand, GGOs with consolidation or other abnormalities (i.e., reticular and/or interlobular septal thickening) involving multiple lobes could be more common in patients with imported infection than in patients with the second-generation infection, resulting in elevated CT density and extent scores in patients with imported infection when compared with patients with second-generation infection. Our findings suggest that patients with imported infection might have more rapid progression of disease and increasing likelihood of mixed bacterial coinfection.^[18–19]^ Based on the comparison of CT density score between groups, we can presume that the COVID-19 in second-generation infected patients could be milder when compared to those with imported infection.

Our study had several limitations. For one thing, a larger sample size of COVID-19 patients is required for further investigation, especially with an emphasis on asymptomatic second-generation patients. For another thing, the semi-quantitative scoring system of disease in this study was based on the typical CT manifestations applied in the expert consensus,^[9]^ the other abnormal findings such as reticulation and interlobular septal thickening did not particularly evaluate, and further modification is required.

## 5 Conclusions

The CT findings of COVID-19 vary according to the routes of infection. Patients with imported infection tend to have more severe CT manifestations, suggesting that CT could accurately evaluate the COVID-19 in the population. Cases with second-generation infection could be manifested as normal finding on the initial CT scan, but may progress to mild abnormalities on follow-up CTs, indicating 2019-nCoV detection via RT-PCR could be essential in the population with high risk of infection. We hope that our findings can help clinicians outside the original district (Wuhan, China) of this disease formulate more accurate and effective prevention and treatment measures.

## Author contributions

**Clinical studies:** Yu-ping Wu, Jin-ming Cao

**Experimental studies/data analysis:** Yu-ping Wu, Jin-ming Cao, Tian-wu Chen, Rui Li

**Guarantor of integrity of the entire study:** Yu-ping Wu, Jin-ming Cao, Tian-wu Chen

**Literature research:** Yu-ping Wu, Jin-ming Cao, Rui Li

**Manuscript editing:** Yu-ping Wu, Jin-ming Cao, Tian-wu Chen, Rui Li, Feng-jun Liu, Yue Zeng, Xiao-ming Zhang, Qi-wen Mu, Hong-jun Li

**Manuscript preparation:** Yu-ping Wu, Jin-ming Cao, Tian-wu Chen, Rui Li

**Statistical analysis:** Yu-ping Wu, Jin-ming Cao, Tian-wu Chen, Rui Li

**Study concepts and design:** Tian-wu Chen, Rui Li, Feng-jun Liu, Yue Zeng, Xiao-ming Zhang, Qi-wen Mu, Hong-jun Li
